# Facilitators and Barriers to Implementing High-Intensity Gait Training in Inpatient Stroke Rehabilitation: A Mixed-Methods Study †

**DOI:** 10.3390/jcm13133708

**Published:** 2024-06-25

**Authors:** Julia Aneth Mbalilaki, Ingvild Lilleheie, Stein A. Rimehaug, Siri N. Tveitan, Anne-Margrethe Linnestad, Pia Krøll, Simen Lundberg, Marianne Molle, Jennifer L. Moore

**Affiliations:** 1 Regional Rehabilitation Knowledge Center, Sunnaas Rehabilitation Hospital, 1453 Nesodden, Norway; julmba@sunnaas.no (J.A.M.); ingvild.lilleheie@sunnaas.no (I.L.); stein.arne.rimehaug@sunnaas.no (S.A.R.); siri.tveitan@sunnaas.no (S.N.T.); anne-margrethe.linnestad@sunnaas.no (A.-M.L.); 2 Department of Nursing and Health Sciences, University of South-Eastern Norway, 3045 Drammen, Norway; 3 Skogli Health and Rehabilitation Center, 2614 Lillehammer, Norway; pia.kroll@skogli.no; 4 Division of Physical Medicine and Rehabilitation, Vestfold Hospital, 3103 Tønsberg, Norway; simelu@siv.no; 5 Indre Østfold Municipality, 1830 Askim, Norway; marianne.molle@io.kommune.no; 6 Institute for Knowledge Translation, Carmel, IN 46082, USA

**Keywords:** implementation science, knowledge translation, translation science, biomedical, gait disorders, neurologic, stroke rehabilitation, physical therapy specialty

## Abstract

(1) **Background**: High-intensity gait training (HIT) is a recommended intervention that improves walking function (e.g., speed and distance) in individuals who are undergoing stroke rehabilitation. This study explored clinicians’ perceived barriers and facilitators to implementing HIT utilizing a mixed-methods approach comprising a survey and exploratory qualitative research. (2) **Methods**: Clinicians (n = 13) who were implementing HIT at three facilities participated. We collected and analyzed data using the consolidated framework for implementation research. Three focus groups were recorded and transcribed, and data were coded and thematically categorized. (3) **Results**: Survey results identified that the facilitators with a strong impact on implementation were access to knowledge/resources and intervention knowledge/beliefs. The only agreed-upon barrier with a strong impact was lack of tension for change. The focus groups resulted in 87 quotes that were coded into 27 constructs. Frequently cited outer setting facilitators were cosmopolitanism and peer pressure, and the only barrier was related to the patient needs. Innovation characteristics that were facilitators included relative advantage and design quality and packaging, and complexity was a barrier. Inner setting facilitators included networks and communication, learning climate, leadership engagement, and readiness for implementation. However, communication, leadership engagement, and available resources were also barriers. Regarding characteristics of individuals, knowledge and beliefs were both barriers and facilitators. In the implementation process domain, common facilitators were formally appointed implementation leaders and innovation participants. Barriers in this domain were related to the patients. (4) **Conclusions**: Clinicians identified many barriers and facilitators to implementing HIT that often varied between facilities. Further research is warranted to deepen our understanding of clinicians’ experiences with HIT implementation.

## 1. Introduction

Across areas of rehabilitation, research studies have demonstrated that the treatment provided to patients is often inconsistent with the best available evidence. Despite an increasing focus on evidence-based practice, rehabilitation clinicians frequently prescribe interventions based on clinical experience or what they learned in school, which is often outdated or unsubstantiated information [1,2,3]. Research indicates that >90% of clinicians choose treatments based on previous education or outdated texts instead of using more recent evidence to guide practice [4]. In a survey of 244 physical therapists, 87% reported that they used evidence to support clinical decision-making <5 times/month, and 33% reported using evidence <2 times/month [5].

Stroke impacts ~15,000 Norwegians annually, and the incidence of strokes among individuals at younger ages (<60 years old) is increasing [6,7]. Many individuals present with persistent lower extremity weakness, impaired coordination, and involuntary motor behaviors [8]. These impairments limit the ability to independently perform many functional tasks, including walking without assistance, rising from a chair, and maintaining balance, all of which limit mobility in the home and community [1,9]. Reduced mobility and decreased physical activity can accelerate the deterioration of cardiovascular and metabolic health, which further limits health and participation [10,11].

One area of stroke rehabilitation research that may have a substantial impact on patient outcomes, cost-effectiveness, and long-term community participation is high-intensity gait training (HIT) [1,12,13,14]. Results from many HIT studies have demonstrated that walking interventions that maximize the number of steps taken and are performed at high aerobic intensities (i.e., >70% maximum heart rate) result in substantial improvements in walking speed, distance, endurance, and walking economy [1,12,15,16,17,18,19]. When implemented into inpatient stroke rehabilitation approximately 4 times a week, patients experience substantially larger gains in walking distance and speed compared to usual care [20,21,22].

Despite these results, the clinical use of HIT is limited in stroke rehabilitation. Observational studies suggest that interventions typically consist of the practice of many different tasks and focus very little on walking practice during inpatient rehabilitation [1,2,23]. Further, aerobic intensities of interventions are rarely achieved, reaching aerobic thresholds <5% of sessions [24] and averaging 30–40% age-predicted HR reserve throughout sessions [24].

Knowledge translation (KT) aims to improve the efficiency and effectiveness of implementing evidence into practice. Focused KT efforts may enhance the implementation of high-intensity gait training (HIT) in stroke rehabilitation. Research suggests that targeting barriers with implementation strategies increases the likelihood of implementing a practice with fidelity (i.e., as it was designed or studied) [25,26,27]. Common barriers to implementing HIT include time, knowledge, skills, beliefs, reimbursement, low priority or competing priorities within the organization, and inadequate resources [28]. Frequently cited facilitators include the inclusion of KT in a clinician’s role, organizational or leadership support, knowledge tools, co-development of the implementation plan, and mentoring [28]. Specific barriers to providing aerobic exercise programs and HIT include concerns for patient safety, comorbidities, cognitive limitations, fatigue, emotional well-being, knowledge, beliefs, adaptability, and available resources [29,30,31].

While providing insight, the published data lack systematic qualitative methods that facilitate a deeper understanding of the barriers. Consequently, this mixed-methods study included a survey and exploratory qualitative research and aimed to explore clinicians’ perceived barriers and facilitators to HIT.

## 2. Materials and Methods

These data were collected as part of an ongoing HIT implementation project in Norway. Two sites, a public hospital with a rehabilitation unit and a private rehabilitation hospital, were participating in a two-phase KT project facilitated by the Regional Rehabilitation Knowledge Center (RKR, Oslo, Norway). Phase one included implementing standardized tests and phase two focused on implementing HIT. While these facilities were participating in a HIT implementation project, no external funding was secured to support the implementation activities. The third site, an inpatient public municipality-based hospital, was educated in HIT using a RKR course and support from local clinicians trained in HIT. This site implemented standardized tests and HIT simultaneously. At all three sites, clinicians were trained in HIT and encouraged to use the intervention. However, they were not required to change practice or implement HIT with fidelity.

The implemented HIT protocol (previously published in the appendix of [16]) included administering balance and gait outcome measures at admission, weekly, and discharge [20,30]. Recommendations for HIT implementation included delivering task-specific walking on a treadmill, overground, and on stairs at least four 1-hour sessions per week [13]. Recommendations also included spending ≥40% of the session at 70–85% of the age-predicted maximum heart rate [20,21,30]. Clinicians who participated in this project were learning HIT and actively implementing HIT, but they were not consistently implementing HIT with fidelity.

### 2.1. Study Design and Population

A mixed-method design with quantitative (survey) and qualitative (focus groups) approaches was employed. Clinicians were eligible if they were full- or part-time employed and worked with patients undergoing stroke rehabilitation and attempting to use HIT in clinical practice. We excluded clinicians who were temporary or contract employees. The Regional Committee for Medical and Health Research Ethics (REK) approved the project (REK ref. no. 2016/873 and 2020/672). All clinicians provided signed informed consent.

### 2.2. Procedure, Data Collection, and Data Analysis

We collected and analyzed survey and focus group data using the consolidated framework for implementation research (CFIR) [32,33]. The original CFIR includes 5 domains and 37 constructs that are associated with effective implementation. The five domains are intervention characteristics, outer setting, inner setting, characteristics of the individuals, and the implementation process. Comprehensive definitions of the domains and constructs are available on the CFIR website [34].

### 2.3. Surveys

To identify the CFIR domains and constructs to explore during the focus group interviews, we used a pragmatic context assessment tool (pCAT) [35,36]. This 14-item questionnaire provides an abbreviated assessment of local facilitators and barriers in a clinical setting. The tool asks clinicians to identify barriers, facilitators, and their potential impact on implementation. We modified questions to focus on HIT and included those related to commonly cited barriers. The survey was translated into Norwegian by one investigator (JAM), a non-native Norwegian speaker fluent in English. A second investigator (SA), a native Norwegian speaker fluent in English, compared the Norwegian and English versions to identify conflicts in meaning for each item. Conflicts were resolved collaboratively, resulting in revisions. To confirm face validity, 3 clinicians took the survey and provided feedback. Through an iterative feedback and revision process, the Norwegian pCAT was finalized.

### 2.4. Focus Groups

We conducted three site-specific focus groups to explore barriers and facilitators identified on the pCAT. Selected CFIR constructs with strong or conflicting effects were explored using questions from the CFIR Interview Guide Tool that was based on the original version of CFIR [37]. Two investigators, JAM (project leader) and SNT (a native Norwegian speaker), ensured homogeneity in the focus group conduct. JAM facilitated the interview (project leader), and SNT (co-worker, a native Norwegian speaker) served as the moderator and note-taker and administered the recording. The interviews started with information about HIT and a review of the recommendations for the implementation of HIT, allowing clinicians to reflect and ask questions. Participants were encouraged to openly discuss barriers and facilitators. Only researchers and participants were present in the 60 min focus groups.

### 2.5. Analysis

Survey data were analyzed using Microsoft Excel 2016. Barriers and facilitators at each site were organized in a matrix/table with rows for barriers and facilitators, columns for statements of agreement, and cells for summarized data. Responses were synthesized and ranked based on majority responses, with constructs categorized as having a mixed effect when responses were distributed across rankings. Visual inspection of the data helped identify constructs with a strong or mixed effect at each site.

Audio-recorded interviews were anonymized and professionally transcribed then entered into NVivo 14 (QSR International, Burlington, MA, USA) for coding. The CFIR codebook and NVivo Project templates available through the CFIR website were used [37]. We used a conventional content analysis with a deductive approach for coding [38]. Quotes were coded into CFIR constructs and rated as barrier (+), facilitator (−), or mixed influence (×). One investigator (JAM) performed the initial coding, which was imported into Microsoft Word for further analysis and rating by other investigators. To ensure rigor and trustworthiness, several steps were taken. Two experienced qualitative researchers (SA and IL) independently reviewed the quotes and codes and resolved conflicts during group discussions. The group translated the codes from Norwegian to English. A fourth person (JM, HIT principal investigator and native English speaker) reviewed the quotes and codes and discussed conflicts with the group. After the codes were finalized, we conducted a thematic analysis by grouping quotes into similar concepts. The groups with the strongest and largest number of quotes were identified as the main themes.

## 3. Results

Thirteen clinicians from three facilities participated. Table 1 describes the clinician demographics.

### 3.1. Survey

The pCAT results are delineated by site in Table 2 and by clinician in the Supplemental Table. Within the clinician-level analysis, some practitioners reported encountering barriers with effects that ranged from weak to strong. However, when the constructs were stratified by site, a predominant pattern emerged wherein most clinicians characterized the constructs as either facilitators or as exhibiting mixed effects, which attributed to conflicting responses within the team. Consequently, only one construct, tension for change, was recognized as a barrier by most clinicians at the two facilities. Across all facilities, the facilitators with a strong effect included access to resources and knowledge and beliefs about the intervention. Additionally, patient needs and resources and adaptability were identified as facilitators, but their impact on implementation varied.

### 3.2. Focus Groups

Eighty-six quotes were coded into twenty-seven constructs and are described in Table 3. The most frequently cited outer setting facilitators were cosmopolitanism and peer pressure, and the only barrier was related to the needs and resources of the patients. Common facilitators related to innovation characteristics included relative advantage and design quality and packaging, and the most common barriers were related to complexity. Frequently reported inner setting facilitators included networks and communication, learning climate, leadership engagement, and readiness for implementation. However, communication, leadership engagement, and available resources were also discussed as barriers. Regarding characteristics of individuals, knowledge and beliefs were discussed as both a barrier and a facilitator. In the implementation process domain, the most common facilitators were formally appointed implementation leaders and innovation participants. Barriers in this domain were related to the patients.

The quotes were categorized into four key themes: being a part of something bigger; leadership and organizational support; readiness to change; and delivering the intervention to patients.

1. Being a part of something bigger

The clinicians experienced a sense of being a part of a macro-level movement. They discussed a facilitator related to HIT as being a “*trend in society*”. At the professional level, the clinicians felt that “*being a part of a professional community that influences progress*” was important. They also experienced a professional connection: “*this community and being a part of a project that gives us a professional community, has been extremely important*” (F3-P3) [+Cosmopolitanism]. Mentoring and support from others who were contributing to this movement helped them overcome barriers and stated that the clinicians are “*there for us (…) there are so many issues along the way in the process, which are not easy to handle alone*” (F1-P1) [+Cosmopolitanism]. Moreover, “*it is also good to know there are other institutions doing the same as us, so that we are not all [alone]*” (F2-P2) [+Cosmopolitanism].

On the other hand, some clinicians experienced barriers, including feeling excluded from the movement. Clinicians reported: “*It is almost a dispute over who gets the patient, so you almost don’t have any patients, and then you lose ownership to the project*” (F2-P4) [−Networks and Communication]. They expressed frustration with the lack of patients with whom to practice or information to contribute to the movement. Some clinicians experienced that others not involved in the movement would criticize their treatments: “*colleagues who do not work on my team, in the stairs, they might say, ‘you push them too hard’*” (3-P3) [−Compatibility, Learning Climate]. When patients received conflicting information about stroke rehabilitation from uninformed individuals, clinicians felt challenged: “*other [patients] are more critical and yes: ‘but my physiotherapist back home are stretching and such (…)’. And then we are saying that: ‘now we are doing it this way’*” (F3-P3) [−Innovation Participants].

2. Leadership and organizational support

The leaders facilitated implementation when they were actively engaged and provided support. “*An important facilitator is the support we have from the management and the head of the physiotherapists*” (F3-P2) [+Leadership Engagement]. Some clinicians felt that delivering the intervention was an expectation which increased their motivation, awareness, and willingness to change: “*It is a clear expectation in this organization, that this is what we want to do, if (the patients) have had a stroke and want to be better at walking, we shall always consider this to the be the best intervention. It’s a clear expectation from the management that this is what we are going to do”* (F1-P2) [+Goals and Feedback]. A strong facilitator was reported when the leader helped in overcoming barriers and prioritized implementation: “*[the team leader] just carries on, if there is a small problem that needs to be solved, we experience that this is prioritized*” (F3-P2) [+Leadership Engagement].

Conversely, clinicians reported barriers that resulted from unsupportive leaders: “*Our leader is not [supportive]… is not enough involved professionally, [the leader] has just run over us*” (F2-P5) [−Leadership Engagement]. Poor planning resulted in feeling unprepared for implementation. “*It is just as if it has been thrown at us, this project, that we should be involved in it*” (F2-P4) [−Innovation Source, Leadership Engagement]. This led to feeling overwhelmed during implementation: “*there were new things, many things we had to take care of*” (F2-P4) [−planning]. Although leadership stated high expectations: “*this is what we want to do*, *consider this as the best intervention*” (F1-P2) [+Goals and Feedback], they did not have implementation goals. “*I do not think we have a goal, other than that we should use it*” (F1-P1) [−Goals and Feedback].

Support from colleagues was recognized as a facilitator: “*we have a lot of support in several professional groups, with some significance in that sense*” (F2-P3) [+Network and Communication]. Open team communication was a facilitator: “*I experience an open dialogue, an open climate, a professional environment around, so that you can discuss certain cases*” (F1-P2) [+Network and Communication, +Learning Climate]. Being a part of a team was also emphasized: “*it is nice to be a group, working together all the time. We bring out the best in each other and challenge each other regarding any questions we might have*” (F3-P1) [+Culture].

In contrast, clinicians experienced a barrier when colleagues were not supportive. “*It can actually be a barrier if you experience critical glances from colleagues. […] that they are actually a little critical of the fact that they hear [patients breathing hard]*” (F3-P3) [−Learning climate].

3. Readiness for change

When clinicians were aware of and confident about the research, they described increased readiness for implementation. “*I think about all the good research behind this, you know, that it works; it has been researched on, so you can be confident”* (F2-P5) [+Evidence Strength and Quality]. Clinicians also indicated readiness to change when a group agreed upon implementing: “*everyone has reasonably agreed; this is what we should do, here, are willing to change*” (F2-1) [+Learning Climate, Individual Stage of Change]. Group readiness motivated clinicians perform extra work to support implementation. “*All of us are highly motivated for this, because it has been necessary to use the small openings in the schedule, and to bring stuff home and work on it over time*” (F3-P2) [+Individual Stage of Change]. Personal beliefs also influenced readiness for change: “*I really do believe in this*. *It is really a great way to exercise*” (F3-P1) [+Knowledge and Beliefs].

However, some clinicians did not feel confident about the intervention due to lack of experience, knowledge, and follow-up: “*lack of confidence might be a barrier; several of us still feel inexperienced and need more weekly cases, and follow one another to learn more*” (F2-P2) [−Self-efficacy]. The physical labor required to provide HIT was a barrier: “*I’m not always that committed, it’s hard to contribute, because I also have some bad knees, sometimes I just have to pay attention to this and transfer [the patients], depending on how my joints are*” (F2-P4) [−Individual Stage of Change]. For some, the beliefs about physical therapy practice conflicted with the intervention and impacted readiness to change: “*The oldest [physiotherapists] working here said that they were glad they should retire because this was completely against all the physiotherapy they have learned*” (F2-P4) [−Knowledge and Beliefs].

4. Delivering the intervention to patients

The clinicians experienced a facilitator when patients “*asked to be referred for more rounds of the same treatment*” (F1-P1) [+Relative Advantage], indicating that some “*patients really like being out of breath; they might not have been [working this hard] in a long time*” (F1-P1) [+Innovation participants]. Patients were inspired when achieving positive outcome measurement results: “*They rather see that progress in the tests; they become more motivated to make an effort even more*” (F2-P3) [+Innovation participants]. When the clinicians observed patient functional improvements, this confirmed the intervention’s value. “*They more quickly became independent in walking, increased walking distance and walking quality, improved balance more quickly. All the patients are so positive; they also get a psychological boost from the training*” (F2-P5) [+Relative Advantage, +Needs and Resources of Patients].

A prominent barrier involved presenting patients with intervention information, as clinicians observed varying levels of understanding and learning ability. “*Some people need a simpler explanation than others do who may want a bit more detail to get a deeper understanding”* (F3-P2) [−Needs and Resources of Patients, Knowledge and Beliefs]. Sometimes the clinicians struggled with explaining HIT to patients: “*They have gotten information, but they might not have understood well enough*” (F1-P4) [−Innovation participants]. These challenges sometimes led to failed sessions: “*so when they start exercising, it becomes too much, sort of, they do not have any experience with how exhausting it is”* (F1-P4) [−Innovation participants].

## 4. Discussion

In this mixed-methods study of the barriers and facilitators to implementing HIT in inpatient stroke rehabilitation, clinicians reported varying barriers and facilitators across three facilities. The only agreed-upon barrier on the pCAT was tension for change, indicating that the clinicians did not perceive the current situation as intolerable or needing to change. The focus groups identified many additional barriers, which demonstrates the need for mixed-methods research to gain a comprehensive understanding of barriers. While some barriers and facilitators were similar across sites, we identified many site-specific variations. These data suggest that identifying local barriers and facilitators may be critical for successful implementation. Four main themes were identified, including being a part of something bigger, leadership and organizational support, readiness for change, and delivering the intervention to patients.

The study’s findings underscore the significance of participating in broader initiatives, as a prominent theme revolved around being a part of something bigger. The advantages of participating in a movement include factors that may cultivate intrinsic motivation, particularly through personal contributions to advancing neurologic physical therapy. This intrinsic motivation may be significant in the context of self-determination theory (SDT), as research suggests a positive association between intrinsic motivation and attitudes, performance outcomes, and organizational commitment [39]. Thus, fostering intrinsic motivation may facilitate a heightened commitment to implementation. Moreover, clinicians gained support, resources, and collaborative assistance through their involvement in the movement, fostering meaningful connections and enhancing the sense of relatedness, a key motivator in SDT [40,41]. A similar theme, “the bigger picture”, was generated from a qualitative study of barriers and facilitators to a successful community of practice [42]. In this study, clinicians emphasized how their contributions enhanced the rehabilitation field and highlighted the benefits of knowledge sharing and networking [42]. Therefore, emphasizing individual contributions to larger societal goals may facilitate implementation. Additionally, fostering connections among individuals at local, national, and international levels can further support implementation efforts.

Leadership and organizational culture may have contributed to the clinicians’ levels of relatedness and autonomy. Some felt supported by leaders and colleagues, which facilitated overcoming barriers and fostering a positive learning environment. Conversely, some clinicians faced challenges related to a lack of engagement and opportunities to contribute to implementation plans. In accordance with SDT, these barriers seemed to diminish autonomy and motivation to actively participate in implementation [39]. KT research suggests that engagement can impact project ownership [43], and an integrated KT approach that engages knowledge users (e.g., clinicians) throughout the project is recommended [44,45]. This approach includes building relationships based on trust, respect, and transparency; shared decision-making; fostering open and responsive communication; and recognizing, valuing, and sharing diverse expertise [46]. An integrated KT approach may enhance the clinicians’ autonomy and relatedness, which may facilitate implementation.

Research indicates that adaptive (responding to change) and proactive (initiating change) performance are related to needs for competence, autonomy, and relatedness [47]. The clinicians who felt confident in the HIT research and a sense of group participation indicated higher levels of intrinsic motivation that led to actions such as working on the project at home. However, other clinicians reported lower competency, knowledge, and conflicting beliefs about the intervention. These quotes arose from a site that reported less leadership support and perceived the project started abruptly. This may be attributed to an incomplete implementation process, which consists of three phases: pre-implementation, implementation, and competency (fidelity) [48]. The pre-implementation phase includes engaging the team and determining readiness, and research emphasizes the critical role of this phase in implementing with fidelity [49]. These results suggest a potential shortfall in this phase at this facility, which may have impacted readiness. Thus, addressing competence, autonomy, and relatedness during the pre-implementation phase may assist in overcoming barriers to successful implementation.

Patients’ responses to the intervention were reported as both facilitators and barriers and may have been impacted by the clinicians’ knowledge and competence. Positive patient outcomes, including hard work, enjoyment, and motivation, were reported as facilitators. Other studies identified that clinicians’ competence in describing care-related procedures was a facilitator for clinicians and patients [50,51]. Conversely, our study identified that lower competency among clinicians when explaining the intervention to patients was a barrier. This underscores the significance of health literacy, which describes people’s knowledge, motivation, and competency in accessing, understanding, and applying health information and making health-related decisions [52]. Collectively, these results highlight the need to enhance competency in delivering clear and usable health information to patients to facilitate implementation [53].

### Study Limitations

We utilized a sample drawn from three Norwegian facilities that were implementing HIT. Consequently, the findings may not represent the perspectives of all clinicians engaged in implementing similar interventions. Although these results may offer insights, additional research is needed to develop a more comprehensive understanding in this area. Throughout the study, the investigators were mindful of their pre-conceptions and strived for an unbiased interpretation of the data, providing a transparent description of the results’ generation. While some investigators were closely involved in HIT implementation studies, the focus group facilitators remained uninvolved in the implementation project operations. The facilitator, a non-native Norwegian speaker, conducted the focus groups, introducing a potential language-related influence on responses. However, a native Norwegian-speaking moderator was present to mitigate language gaps and enhance communication. Furthermore, the focus groups may have prevented some clinicians from openly sharing their feelings. To address this limitation, the facilitator and moderator actively collaborated with the clinicians to foster an open environment. This study used the original CFIR; a revised CFIR was published after we completed data collection [54]. Future studies should incorporate the revised CFIR. Despite these limitations, this study contributes valuable insights to the understanding of HIT implementation.

## 5. Conclusions

In this mixed-methods study, clinicians at three facilities identified several barriers to, and facilitators of, implementing HIT, which varied by facility. In the survey, the frequently identified facilitators with a strong effect included access to knowledge and resources and knowledge and beliefs about the intervention, whereas the only frequently identified barrier among the facilities was tension for change. The focus groups identified many facilitators, with cosmopolitanism being the most cited facilitator. Common barriers were related to the patients and knowledge and beliefs. Four themes emerged, including the bigger picture, leadership and organizational support, readiness for change, and delivering the intervention to patients.

## Figures and Tables

**Table 1:** Characteristics of the clinicians and facilities.

Characteristics of the Clinicians				
Variables	Facility 1	Facility 2	Facility 3	Total
Sex				
Female	4	3	2	9
Male	1	2	1	4
Age (years)				
20–29	1	1		2
30–39	3		1	4
>40	1	4	2	7
Work experience (years)				
<5	2	1		3
5–10	3			3
11–15		1		1
>15		3	3	6
Approximate stroke patients seen in a week per clinician				
>10 patients				
5–9 patients	2	2	1	5
1–4 patients	3	3	2	8
<1 patients				
Approximate number of stroke patients treated with HIT per week				
>10 patients				
5–9 patients	2	1		3
1–4 patients	2	3	3	8
<1 patient	1	1		2
Experience with providing HIT				
Beginner	3	2	2	7
Average	2	2	1	5
Expert		1		1
Employment Setting				
Hospital		5		5
Private rehabilitation institute			3	3
Municipality	5			5
Profession				
Physiotherapist	4	3	3	11
Occupational therapist	1			1
Sport therapist		2		2
Number of Patients with Stroke per Year				
	Facility 1	Facility 2	Facility 3	
Approximate number of patients with stroke per year	70	120	105	

**Table 2:** Pragmatic context assessment tool (pCAT) results. F 1 = Facility 1; F 2 = Facility 2; F 3 = Facility 3.

	Barriers with a Strong Effect	Barrier with a Weak Effect	Neutral	Facilitator with a Strong Effect	Facilitator with a Weak Effect	Mixed Responses
1. People here regularly seek to understand the needs of patients and make changes to better meet those needs. Patient Needs and Resources				F 1 F 2	F 3	
2. I have open lines of communication with everyone needed to make the change. Networks and Communications			F 2			F 1 F 3
3. I have access to data to help track changes in outcomes. Goals and Feedback (or Reflecting and Evaluating depending on context/phase)				F 2	F 1	F 3
4. The implementation of high-intensity gait training is aligned with leadership goals. Relative Priority			F 2	F 1 F 3		
5. The implementation of high-intensity gait training competes with other projects that require resources in my facility. Relative Priority			F 1 F 3	F 2		
6. The implementation of high-intensity gait training is aligned with clinician values. Compatibility			F 2	F 1 F 3		
7. The implementation of high-intensity gait training is compatible with existing clinical processes. Compatibility			F 1 F 3	F 2		
8. The structures and policies in place here enable us to successfully implement high-intensity gait training. Compatibility; Structural characteristics				F 1 F 3		F 2
9. We have sufficient space to implement high-intensity gait training. Available Resources				F 1 F 3		F 2
10. We have sufficient time dedicated to implement high-intensity gait training. Available Resources			F 1 F 3	F 2		
11. We have other needed resources to implement high-intensity gait training (staff, money, supplies, etc.). Available Resources				F 3		F 1 F 2
12. People here see the current situation (i.e., usual care) as intolerable and that the change is needed. Tension for Change		F 2 F 3				F 1
13. People here see the advantage of implementing high-intensity gait training versus an alternative change. Relative Advantage			F 2 F 3			F 1
14. Higher level leaders are committed, involved, and accountable for implementation of high-intensity gait training. Leadership Engagement			F 1 F 3			F 2
15. Leaders I work with most closely are committed, involved, and accountable for the implementation of high-intensity gait training. Leadership Engagement				F 1 F 3		F 2
16. The high-intensity gait intervention can be implemented in a way that meets my patient’s needs. Adaptability				F 1 F 2	F 3	
17. The high-intensity gait intervention can easily be implemented in my own practice. Complexity			F 3	F 1 F 2		
18. I have the resources and materials that I need to successfully implement high-intensity gait training. Design Quality and Packaging				F 1 F 3		F 2
19. High-intensity gait training is considered an important intervention to implement by the health services (i.e., payers). Peer pressure, external policies and incentives			F 1 F 3			F 1 F 2
20. The culture of my organization will support the implementation of high-intensity gait training. Culture			F 3	F 1		F 2
21. I have access to the training and mentoring that I need to successfully implement high-intensity gait training. Access to knowledge and information				F 1 F 2 F 3		
22. I am confident that I will be able to successfully use high-intensity gait training with my patients. Self-efficacy				F 2 F 3		F 1
23. Clear implementation goals for high-intensity gait training have been identified. Reflecting and evaluating			F 1	F 3		F 2
24. Clinicians and leaders who will champion and lead this change have been identified in my department. Opinion leaders, formally appointed internal implementation leaders, and champions				F 2 F 3		F 1
25. I believe high-intensity gait training will result in better patient outcomes than my usual care interventions. Knowledge and Beliefs about the Intervention				F 1 F 2 F 3		

**Table 3:** Facilitator(s) and barrier(s) according to CFIR domain and constructs.

(**a**) **Consolidated Framework for Implementation Research (CFIR) Domain—Innovation Characteristics**		
Construct–Impact	Description	Quotes
Innovation Source—Barrier(s)	Not offered a choice to participate in the project	*It is just as if it has been thrown at us, this project, that we should be involved in it. F2-P4 (−)*
Strength and Quality—Facilitator(s)	Aware of evidence that supports the intervention	*I think about all that good research behind this, you know, that it works; it has been researched on, so you can be confident. F1-FP5 (+)* *I think that’s very good, and it’s systematized so you know it works, that there are research showing that it is effective, being able to tell them this, that’s good, I think. F3-P2 (+)*
Relative Advantage—Facilitator(s)	Patient and caregivers’ desire to perform HIT	*Both relatives and the patients ask to be referred for more rounds of the same treatment. F1-P1 (+)*
	Clinicians’ observations of impact of HIT on patients	*Yes, they become more quickly independent in walking, and they get increased walking distance and walking quality. Many of them also gets better balance more quickly. F2-P5 (+)* *All the patients I have worked with are so positive, and it seems like they also get a psychological boost from the training. Exercising with high intensity, and then you get that in addition. F2-P5 (+)*
	Belief the work to use HIT is worth the effort	*Even though this is complex, the trade-off is worth it. F3-P2 (+)*
Relative Advantage—Barrier(s)	Costs related to delivering, considering patient volume	*To invest is a very large investment for a small institute. So far, I have not had enough patients for it to be defendable/justifiable/proper… In terms of resources, you could say that it is a barrier there. F1-P3 (−)*
Adaptability—Facilitator(s)	HIT can use commonly available equipment	*I do think it is important to tell both the patient and next therapist that it doesn’t have to be so complicated. As you said, you don’t need a treadmill, you can do a whole lot only using a stairway, walking outside, walking inside, finding some obstacles, it does not have to be expensive. There are some things you do need, to ensure safety, but anyway, there are several things you can do that is so much closer to HIT than what has been done in the past using inexpensive stuff, but you have to have the knowledge. F3-P3 (+)*
Adaptability—Barrier(s)	Scheduling HIT sessions	*And then it is about the time, we can count the minutes used on a “[HIT]” patient, and maybe we use fifteen minutes more than on a patient not included in the “[HIT]” project, but you can’t just put them in anywhere in your calendar, because you can’t do high-intensity gait training right after the patients breakfast, and maybe they have to have patient education with the doctor or other things they have to do, so we have to make sure that they get some rest both before and after, that’s also a barrier. F3-P2 (−)*
Complexity—Barrier(s)	Many tasks required in HIT protocol	*Starting to follow protocols on measuring blood pressure, (…) all the things you do not have to do when carrying out a training session to know how the patient are doing, how is the blood pressure, as we are told to have an eye on…, it’s been difficult for people to do all this. F2-P5 (−)*
	Requirements for achieving fidelity	*I think it is a barrier with some of the patients when I’m not able to increase their pulse above 75%, and you have to have them there for 40% to be able to call it “[HIT]”, and it’s supposed to be like this for so and so long. It has also been a barrier that, everyone should have one-hour treatment, and you cannot be doing anything else. F3-P2 (−)*
Design Quality and Packaging—Facilitator(s)	In-person workshop on HIT	*It was something else being at [the course] this time. It was exciting to see how they pushed, because I got a much better impression being there, than looking upon it theoretically or having online discussions. F1-P2(+)*
	Accessibility of knowledge and variability in educational content	*Especially, also because they [clinicians trained in HIT] have a kind of buffet (…). You get very concrete examples. Even if you only see it on video, that’s one thing, but when you are in the room and watching it, that enriches the range of what you see. F1-P5 (+)*
	Continuous access to educational information in Norwegian	*All the courses we have gone through are available for us every day. Therefore, we can look on them, and we have graphs that clearly shows us that if this is the barrier, we can try this intervention (…). Now everything that was previously only in English is also in Norwegian, and the coursers and the graphs and everything is there, so it is possible to obtain knowledge. F2-P5 (+)*
(**b**) **CFIR Domain—Outer Setting**		
Construct–Impact	Description	Quotes
Needs and Resources of Those Served by the Organization—Barrier(s)	Patients’ health and fitness status	*Many of them have chronic ailments, sort of, and are thereby deconditioned compared to if they were admitted directly after this happened, they might have had better fitness condition. They have pretty low fitness capacity, the ones we meet. F1-P2 (−)*
	Side effects of exercise	*We experienced, now recently, with the last patient I have worked with lately, that he complained about hip pain on his healthy side, hip and knee maybe. They are worn out there. F2-P2 (−)*
	Patients’ ability to understand information about HIT	*Some people need a simpler explanation than others do who may want a bit more detail to get a deeper understanding. F3-P2 (−)*
Needs and Resources of Those Served by the Organization—Facilitator(s)	Observation of positive impact of HIT on patients.	*All the patients I have worked with are so positive, and it seems like they also get a psychological boost from the training. Exercising with high intensity, and then you get that in addition. F2-P5 (+)*
Cosmopolitanism—Facilitator(s)	Good peer collaboration and mentoring	*It is great that if we have any questions, they [other clinicians trained in HIT] are there for us. After all, they are more experienced than we are. In addition, there are so many issues along the way in the process, which are not easy to handle alone. That is why it is nice that someone is there to ask. They are so eager to help. F1-P1 (+)*
	Participating in a project with other institutions and identifying role models	*It is also good to know there is other institutions doing the same as us, so that we are not all [alone]…, yes. And that there is someone like [other facility that successfully implemented HIT], further ahead and, that we have something to aim for and get better. F2-P2 (+)*
	Gaining a positive reputation in the professional community by participating	*One thing is the formality, but the physiotherapy community in Norway is not that big, so the word will go around about what we are doing here, and this will influence positive on our reputation. F3- P2 (+)*
	Being a part of a community that is advancing a profession	*I do not think it is the project alone, but a combination of things. But being a part of a professional community that influence progress, and this community, and being a part of a project that gives us a professional community, that has been extremely important, because we actually have…, I’m sure we have recruited a lot of patients that way. F3-P3 (+)*
	Receiving patient referrals because the facility is delivering HIT	*Yes, we are working to get information out about the offer we have, constantly/continuously. We have had some [public relations] rounds in the past, where we have travelled around in some places and talked/informed to the people about our offer. It has been a few years now since we did that, because of the pandemic. However, we have also given information to/informed municipalities and [general practitioners], for example here in the area. It is also important to spread the information about our offer in member’s magazines, for example in Stroke and Aphasia and the [National Association for Heart and Lung Disorders]. I know we have had more referrals/inquiries because people have read about it, or the rumors have spread. F3-P3 (+)*
Peer pressure—Facilitator(s)	Learning about others who successfully implemented HIT	*I remember that we were at that rehabilitation conference in Kristiansand, when [clinicians from a facility that implemented HIT] presented this for the first time, maybe, so it must be 5 or 6 years ago. F2-P3 (+)*
	Belief that HIT should be delivered to “keep up” in the field	*But then I remember us talking to [an advisor] in a meeting, that if we were to be in the ball game with everything going on in rehabilitation and stroke rehabilitation, we can’t say no to this. F2-P2 (+)*
	Gaining a positive reputation because of delivering HIT	*One thing is the formality, but the physiotherapy community in Norway is not that big, so the word will go around about what we are doing here, and this will influence positive on our reputation. F3-P2 (+)*
	Belief that delivering HIT will help secure future contracts in the health system	*Since we are a private rehabilitation centre, we are not a competitor to [other hospitals implementing HIT], but I do want to say that high-intensity gait training is an absolute advantage for us. F3-P3 (+)* *However, we cannot stop here you know, because it will be a continuous process forward, and we know that sooner or later there will be another contract competition. Then we can highlight that we can offer this intervention, and if this is the trend in the society, or if more people get their eyes open to the fact that this is important, I do think that this can be crucial whether we will be able to keep contract or not. F3-P3 (+)*
External Policy and Incentives—Facilitator(s)	Belief that delivering HIT will help secure future contracts in the health system	*We have a contract to bear in mind, and that’s kind of a barrier, and also maybe a facilitator since we have to move on because we don’t have time to wait, but that might also be a barrier because it limits our opportunities. F3-P3 (+)*
	Belief that offering HIT may influence others to refer patients to the facility	*Both in terms of winning future contracts (with the health authorities), but also when it comes to recruiting patients from the hospitals, because if they know that we offer good treatment, they will recommend us to their patients, and it is these recommendations that will influence where the patients will decide to go for rehabilitation after discharged from the emergency department in the large hospitals in Oslo, where from we usually receive patients. F3-P3 (+)*
(**c**) **CFIR Domain—Inner Setting**		
Construct–Impact	Description	Quotes
Structural Characteristics—Barrier(s)	Competing priorities related to patients and work responsibilities	*It has been a barrier, the way we are organised, because we have other patient groups we must alternate, and we have responsibilities that makes it impossible to follow up on everything we are supposed to do and prioritise. F2-P5 (−)*
Networks and Communications—Facilitator(s)	Importance of open communication and collaboration	*I experience an open dialogue, but sometimes we disagree. I think it’s important to be open for discussion if you aren’t certain. Between those I work closest with, I mean we have an open climate. F1-P2 (+)* *I think it is important to have a professional environment around, so that you can discuss certain cases. F1-P2 (+)*
	Other professional involvement and effect of HIT on different body functions	*Therefore, it is fun when we have engaged neuropsychologists who also come up with things in relation to plasticity in the brain and heart rate increase. Is not it, it has an effect on more things than (indistinct) then, that is, in relation to physical health, cognition not least. Therefore, it is a bit of a win-win on several levels, it is not just another function. Moreover, I think we have a lot of support in several professional groups, with some significance in that sense. F2-P3 (+)*
Networks and Communications—Barrier(s)	Lack of patients	*We have had quite few stroke patients for a while, so we don’t get stroke patients to work with [doing HIT], it is almost a dispute over who gets the patient, so you almost don’t have any [inaudible] patients, and then you lose ownership to the project for a while. F2-P4 (−)*
	Lack of information, engagement, and feeling excluded	*I do feel that we sometimes aren’t that included, a lot of information never reaches us, and due to this I stop following and loses my engagement (…). We do not get enough information, we don’t have any papers, we don’t have sufficient experience. And then we are all asked to go through this and this. Therefore, sometimes, I do feel a little bit on the side of the project, from my point of view. This might be some criticism, but it is a little bit like that. F2-P4 (−)*
Culture—Facilitator(s)	Strong group support and collaboration	*I think it is nice to be a group; we are 3–4 working together all the time. We bring out the best in each other and challenge each other regarding any questions we might have. I do think, going through this all alone would have been tough. The fact that we are a group, and… F3-P1 (+)*
Implementation Climate, Compatibility—Barrier(s)	Negative comments from colleagues	*One thing that came to my mind—when I meet, colleagues who do not work on my team, in the stairs, they might say, “you push them too hard”, and that might almost be a barrier. F3-P3 (−)*
Implementation Climate, Goals and Feedback—Facilitator(s)	Leaders’ expectation on innovation	*It is a clear expectation in this organisation, that this is what we want to do, if they (the patients) have had a stroke and wants to be better at walking, we shall always consider this to the be the best intervention. It’s a clear expectation from the management that this is what we are going to do. F1-P2 (+)*
Implementation Climate, Goals and Feedback—Barrier(s)	Lack of implementation goal	*I do not think we have a goal for it in the institute other than that we should use it as much as possible when we see that it is the right measure/intervention. F1-P1 (−)*
Implementation Climate, Learning Climate—Facilitator(s)	Openness to trying new things	*I have the same impression, that we are very open to try new things. Always onto new research and interventions. F1-P4(+)*
	Open communication	*I experience an open dialogue, but sometimes we disagree. I think it’s important to be open for discussion if you aren’t certain. Between those I work closest with, I mean we have an open climate. F1-P2 (+)*
	Group consensus to implement	*Also, everyone is reasonably agreed on that this is a good thing; this is what we should do. None of us healthcare professionals are resisting this change. F 2-P1 (+)*
Implementation Climate, Learning Climate—Mixed	Criticism from colleagues	*However, it can actually be a barrier if you experience critical glances from colleagues. It could be positive, it could be praise, you are really good, but it could also simply be that they are actually a little critical of the fact that they hear their breath go away. F3-P3 (X)* *One thing that came to my mind—when I meet, colleagues from my team in the stairs, they might say, “you push them too hard”, and that might almost be a barrier. F3-P3 (−)*
Readiness for Implementation, Leadership Engagement—Facilitator(s)	Leader support	*Our managers have been very supportive of us going to [other facilities] for follow-up and such, so it has been arranged from the top of the organization to go there. F1-P1 (+)* *Time is a barrier, but it doesn’t feel like a problem, but it could have been, if we didn’t have support from the management, or if we weren’t motivated, for example. F3-P2 (+)* *I think that an important facilitator is the support we have from the management and the head of the physiotherapists. F3-P1 (+)* *I do think that the job [the team leader] has done, we do have to boast about the team leader for the stroke team, who has worked a lot to get routines and procedures in place, everything from forms and contact with [the external facilitator], and it would not have gone as well without [the team leader]. F3-P2 (+)* *In addition, [the team leader] just carries on, if there is a small problem that needs to be solved, we experience that this is prioritised. Now, this is what we do and focus on, so that is a facilitator to solve any barrier that may arise, actually. F3-P2 (+)*
Readiness for Implementation, Leadership Engagement—Barrier(s)	Lack of leadership support and engagement	*The management has been supportive, but our leader is not… is not enough involved professionally, she has just run over us, you know. F2-P5 (−)*
	Was not provided with choice to participate.	*It’s just like it’s been thrown at us, this project, that we should be involved in it. F2-P4 (−)*
Readiness for Implementation, Available Resources—Facilitator(s)	Availability of the equipment	*Therefore, that is also something we found we had to find a solution for it. It. Also, that all the equipment, now we have shelves so that they are close to where we are in the training hall, that the equipment is easily accessible. That we have more braces. F3-P3 (+)*
	Assistance in compiling the data	*Mostly, we talk about facilitators, but having control of all the data we collect—that is a barrier, but we do have a great facilitator in [the researcher]. He has full control of the statistical programs and plots all the data, so we do not have to spend any time on that ourselves. We register on paper when we finish the patient and then we review it, and give it to [the researcher], who plots it. F 3-P3 (+)* *And then we have [the researcher] who takes care of the data, it’s great, we just hand it over to him and he plots and do all the work connected to that, so we don’t have to do this ourselves. F3-P1 (+)* *We do the practical thing, but [the researcher] plots a thousand numbers/figures on each patient. F3-P3 (+)*
Readiness for Implementation, Available Resources—Barrier(s)	Lack of therapists on staff	*We do not always feel that we have enough resources, regarding therapists. F1-P3 (−)*
	Lack of the patients and equipment	*To invest is a very large investment for a small institute So far, I have not had enough patients for it to be defendable/justifiable/proper… In terms of resources, you could say that it is a barrier there. F1-P5 (−)*
Readiness for Implementation, Access to Knowledge and Information—Facilitator(s)	Attending in-person workshop	*It was something else being at [a facilitate that implemented HIT] this time. It was exciting to see how they pushed, because I got a much better impression being there, than looking upon it theoretically or having online discussions. F1-P2 (+)*
	Access to and variability in education resources	*Especially, also because they [colleagues trained in HIT] have a kind of buffet (…). You get very concrete examples. Even if you only see it on video, that’s one thing, but when you are in the room and watching it, that enriches the range of what you see. F2-P3 (+)*
	Taking educational course as a group	*Another thing you mentioned as a barrier, regarding knowledge, that was a barrier for us in the beginning, I would say, but then we joined the gait course on the “knowledge translation” page, which we worked on rather systematically, both individually and as a group. My opinion is that this has been a great course, which has given us the confidence we maybe needed to know what to do, and with a specific focus on which subcomponents the patient needs to practice on, how to balance both the intensity and the number of repetitions ad the focus on subcomponents, I feel that this has been an extremely important course, giving us the right foundation, even though we still are in the beginning of this. F3-P3 (+)*
Readiness for Implementation, Access to Knowledge and Information—Barrier(s)	Education in different language, Lack of ability to practice HIT after education	*It was quite intensive, and in English, and it has been a while, and when there is a period in between when you haven’t been at work and working with intensive gait training, for me, the knowledge seems to fade a little bit. F2-P1(−)*
(**d**) **CFIR Domain—Characteristics of Individuals**		
Construct–Impact	Description	Quotes
Knowledge and Beliefs about the Innovation—Facilitator(s)	Belief in the intervention Confidence in delivering HIT and communicating with colleagues	*I really do believe in this. It is really a great way to exercise.* *F3-P1(+)* *I have more much more faith in that. I also believe that you should train them with high intensity and heart rate. It’s somewhat like heart training, you combine those two. F3-P1 (+)* *We have become familiar with which intensity zones the patients have had during the session and it has gone well. Thereby you are able to say something about where their normal pulse is and then maybe the physiotherapist back home also will be more confident that this way of exercising is possible to carry out. F3-P2 (+)*
Knowledge and Beliefs about the Innovation—Barrier(s)	Explaining the intervention in an understandable manner	*Moreover, maybe some people need a simpler explanation than others do who may want a bit more detail to get a deeper understanding. F3-P2 (−)*
	Conflicting beliefs about clinical practice	*The oldest (physiotherapists) working here said that they were glad they should retire because this was completely against all the physiotherapy they have learned. That I remember! F2-P4 (−)* *Sometimes I do think we have too much focus on whether we should do this or that, instead of just doing it. I wish that we in the beginning just started practising high intensity and more steps, and then figuring out the details along the way. F2-P4 (−)*
Self-efficacy—Barrier(s)	Lack of confidence	*I also think lack of confidence might be a barrier. I think that several of us still feel a little inexperienced and needing more, maybe take up again weekly cases, and maybe follow one another to learn more. F2-P2 (−)*
Individual Stage of Change—Facilitator(s)	Openness to change	*I do feel that all of us, who are working here, are willing to change. F1-P3 (+)*
	Highly motivated clinicians working as a team	*I do experience that all of us is highly motivated for this, and if we had not been, it would have been difficult to pull through, because it has been necessary to use the little gaps in the timetable, and to bring stuff home and work on it over time. F3-P2 (+)*
Individual Stage of Change—Barrier(s)	Physical workload for the physical therapist	*What I do know, is that I’m not always that committed, and I also have some bad knees, and it’s not so great when we don’t function that well, then it’s hard to contribute, sometimes I just have to pay attention to this and transfer them (the patients), depending on how my joints are, and that’s just the way it is. F2-P4 (−)*
(**e**) **CFIR Domain—Process**		
Construct–Impact	Description	Quotes
Planning—Barrier(s)	Lack of planning	*I feel that this has been thrown onto us without a sufficient plan, and it was a lot… After every meeting, there were new things, and suddenly there was a lot of things we had to take care of. What we are doing now is balanced and calm, but the path here has been long and messy, I think. F2-P4 (−)*
Engaging, Formally Appointed Implementation Leaders—Facilitator(s)	Leadership support	*Likewise, I think the work [the team leader] has done, we should be allowed to brag/boast/highlight about as a stroke team leader and has worked a lot to put routines and procedures in place here. Everything from forms and the contact with [the external facilitator], it would not go very well in the same way at all, without [the team leader]. F3-P2 (+)* *In addition, [the team leader] just intensified/drives on, if it’s a small matter that needs to be resolved, we feel that we have always put it here/on the table first. Now this is what we are working on, and that is what we are focusing on, so it will be a facilitator for to solve any barriers that may arise in fact. F3 P2 (+)*
Engaging, Formally Appointed Implementation Leaders—Barrier(s)	Lack of patients and poor distribution of patients	*I think [the opinion leader] had many [patients], it is not unnatural, I do not disagree with that, but it means that the rest of us have lost some ownership in it. F1-P3 (−)*
Engaging, External Change Agents—Facilitator(s)	Referrals for high-intensity gait training	*After all, we receive relatively/quite few inquiries from [general practitioners] about high-intensity gait training, so it is rather the other way around. The doctors who have heard about high-intensity gait training are the ones who have received an e-link from us that is it enough. F1- P2 (+)*
Engaging, External Change Agents—Innovation Participants, Facilitator(s)	Patients enjoy HIT	*There are also patients that really like being out of breath; they might not have been in a long time, being really worn out. Also, in other settings than gait training, they become really worn out. That is positive. F2-P1 (+)*
	Motivation from test results	*They rather see that progress in the tests; they become more motivated to make an effort even more. F2-P3 (+)*
Engaging, External Change Agents—Innovation Participants, Barrier(s)	Patients lack understanding about the intervention	*The patients are often not prepared for how much effort is required for them to achieve the intensity and the frequency you mentioned earlier. A lot of them take for granted that there will be more breaks along the way, which they should be less active. They get tired, starts complaining; want to stop before we actually are done. F1-P4 (−)* *Optimism on their own behalf maybe [patients], overestimating their own capacity, or they hear what you say, but in their own translation, they might think that it probably is not that heavy. There is a lot left to interpretation, so you can be as clear as you like, without this necessarily being perceived the same way. F1-P1 (−)* *Ok, they have gotten information, but they might not have understood this information well enough. We give them expectations that we will keep going for so and so long, so when they start exercising, it becomes too much, sort of, they do not have any experience on how exhausting it is. F1-P4 (−)*
	Conflicting information that patients received about the content of physical therapy	*And some [patients], they swallow it all, sort of, they don’t need that much explanation, but just do as we…, but others are more critical and yes: “but my physiotherapist back home are stretching and such (…).” And then we are saying that: “now we are doing it this way”. To convey new knowledge to the patient. F3-P3 (−)*
Executing—Barrier(s)	Lack of focus during implementation	*It has been a challenge regarding that it has been a little too much back and forth, I totally agree that we have lost track now and then. How to get back on track, and eventually we have. F2-P5 (−)*
	Lack of commitment, information, and treatment fidelity	*There have been some challenges with implementation, getting everyone equally committed, having enough information about the project, and pushing the patients hard enough to actually do what they are supposed to do. F2-P5 (−)*

## Data Availability

The data that informed this article were not stored in a public database due to local restrictions related to storing of research data.

## Supplementary Materials

The following supporting information can be downloaded at: https://www.mdpi.com/article/10.3390/jcm13133708/s1, Supplemental Table. Consolidated Framework for Implementation Research Barrier Buster Tool (CFIR-BBT).
