# Supplementary material for: Facilitators and Barriers to Implementing High-Intensity Gait Training in Inpatient Stroke Rehabilitation: A Mixed-Methods Study
Source: J Clin Med. 2024 Jun 25;13(13):3708. doi: 10.3390/jcm13133708 (PMC11242475; doi:10.3390/jcm13133708)
Supplement: Supplementary file 1 [file jcm-13-03708-s001.zip › jcm-3034890-supplementary.pdf]

Supplemental Table. Pragmatic Context Assessment Tool (pCAT) Results – Combined Individual Clinician Responses					
	**Barriers with a strong effect**	**Barrier with a weak effect**	**Neutral**	**Facilitator with a strong effect**	**Facilitator with a weak effect**
1. People here regularly seek to understand the needs of patients and make changes to better meet those needs. Patient Needs & Resources	1	0	2	8	2
2. I have open lines of communication with everyone needed to make the change. Networks & Communications	2	1	5	4	0
3. I have access to data to help track changes in outcomes. Goals & Feedback (or Reflecting & Evaluating depending on context/phase)	0	0	1	9	2
4. The implementation of high intensity gait training is aligned with leadership goals. Relative Priority	0	0	4	7	1
5. The implementation of high intensity gait training competes with other projects that require resources in my facility. Relative Priority	3	0	5	3	1
6. The implementation of high intensity gait training is aligned with clinician values. Compatibility	1	0	4	8	0
7. The implementation of high intensity gait training is compatible with existing clinical processes. Compatibility	0	1	5	4	2
8. The structures and policies in place here enable us to successfully implement high intensity gait training. Compatibility, Structural characteristics	2	0	1	7	2
9. We have sufficient space to implement high intensity gait training. Available Resources	1	0	4	6	1
10. We have sufficient time dedicated to implement high intensity gait training. Available Resources	1	1	4	5	0
11. We have other needed resources to implement high intensity gait training (staff, money, supplies, etc.). Available Resources	4	0	4	4	0
12. People here see the current situation (i.e. usual care) as intolerable and that the change is needed. Tension for Change	4	6	2	2	0
13. People here see the advantage of implementing high intensity gait training versus an alternative change. Relative Advantage	1	1	8	5	0
14. Higher level leaders are committed, involved, and accountable for implementation of high intensity gait training. Leadership Engagement	1	2	8	1	0

Supplemental Table. Pragmatic Context Assessment Tool (pCAT) Results – Combined Individual Clinician Responses					
	**Barriers with a strong effect**	**Barrier with a weak effect**	**Neutral**	**Facilitator with a strong effect**	**Facilitator with a weak effect**
15. Leaders I work with most closely are committed, involved, and accountable for the implementation of high intensity gait training. Leadership Engagement	1	1	3	7	0
16. The high intensity gait intervention can be implemented in a way that meets my patient's needs. Adaptability	0	0	1	9	2
17. The high intensity gait intervention can easily be implemented in my own practice. Complexity	0	1	4	7	0
18. I have the resources and materials that I need to successfully implement high-intensity gait training Design Quality and Packaging	1	1	2	7	1
19. High intensity gait training is considered an important intervention to implement by the health services (i.e. payers). Peer pressure, external policies and incentives	0	1	7	3	1
20. The culture of my organization will support the implementation of high-intensity gait training. Culture	1	0	6	6	0
21. I have access to the training and mentoring that I need to successfully implement high-intensity gait training. Access to knowledge & information	0	0	1	11	0
22. I am confident that I will be able to successfully use high-intensity gait training with my patients Self-efficacy	0	0	1	11	0
23. Clear implementation goals for high-intensity gait training have been identified. Reflecting and evaluating	1	2	4	6	0
24. Clinicians and leaders who will champion and lead this change have been identified in my department. Opinion leaders, and formally appointed internal implementation leaders, and champions	0	0	3	8	1
25. I believe high-intensity gait training will result in better patient outcomes than my usual care interventions. Knowledge & Beliefs about the Intervention	0	0	2	9	0
